# A novel bacterial symbiont in the nematode *Spirocerca lupi*

**DOI:** 10.1186/1471-2180-12-133

**Published:** 2012-07-05

**Authors:** Yuval Gottlieb, Eran Lavy, Meira Kaufman, Alex Markovics, Murad Ghanim, Itamar Aroch

**Affiliations:** 1 Koret School of Veterinary Medicine, The Hebrew University of Jerusalem, P.O.B 12, Rehovot, 76100, Israel; 2 Kimron Veterinary Institute, P.O.B 12, Bet-Dagan, 50250, Israel; 3 Department of Entomology, Agricultural Research Organization, Volcani Center, P.O.B 6, Bet-Dagan, 50250, Israel

**Keywords:** Spirocercosis, *Comamonas*, Canine, Vector-borne helminthic diseases

## Abstract

**Background:**

The parasitic nematode *Spirocerca lupi* (Spirurida: Thelaziidae), the canine esophageal worm, is the causative agent of spirocercosis, a disease causing morbidity and mortality in dogs. *Spirocerca lupi* has a complex life cycle, involving an obligatory coleopteran intermediate host (vector), an optional paratenic host, and a definitive canid host. The diagnosis of spirocercosis is challenging, especially in the early disease stages, when adult worms and clinical signs are absent. Thus, alternative approaches are needed to promote early diagnosis. The interaction between nematodes and their bacterial symbionts has recently become a focus of novel treatment regimens for other helminthic diseases.

**Results:**

Using 16S rDNA-based molecular methods, here we found a novel bacterial symbiont in *S. lupi* that is closely related to *Comamonas* species (Brukholderiales: Comamonadaceae) of the beta-proteobacteria. Its DNA was detected in eggs, larvae and adult stages of *S. lupi*. Using fluorescent *in situ* hybridization technique, we localized *Comamonas* sp. to the gut epithelial cells of the nematode larvae. Specific PCR enabled the detection of this symbiont's DNA in blood obtained from dogs diagnosed with spirocercosis.

**Conclusions:**

The discovery of a new *Comamonas* sp. in *S. lupi* increase the complexity of the interactions among the organisms involved in this system, and may open innovative approaches for diagnosis and control of spirocercosis in dogs.

## Background

Vector-borne helminthic diseases, such as onchocerciasis and lymphatic filariasis, are major human diseases in endemic areas. Novel treatment approaches have been recently focusing on the interaction between the causative helminth agent and its bacterial symbiont. Consequently, antibiotics, such as doxycycline, are used instead of, or with, anti-helminthic drugs for treatment [1,2]. However, because of difficulties in application, various bacterial targets are constantly studied [3]. This approach has also been adopted in veterinary helminthic diseases, such as bovine onchocerciasis and canine heartworm disease [4-6].

Spirocercosis is a vector-borne helminthic disease, mostly affecting carnivores, especially canids [7,8]. It is caused by the esophageal nematode *Spirocerca lupi* (Spirurida: Thelaziidae) that has a wide distribution, but is mostly prevalent in warm, humid areas. The exact annual number of dogs affected annually worldwide has never been assessed. However, the disease has a wide distribution in the Mediterranean basin, Africa, Central and South America [9].

**Figure 1:**
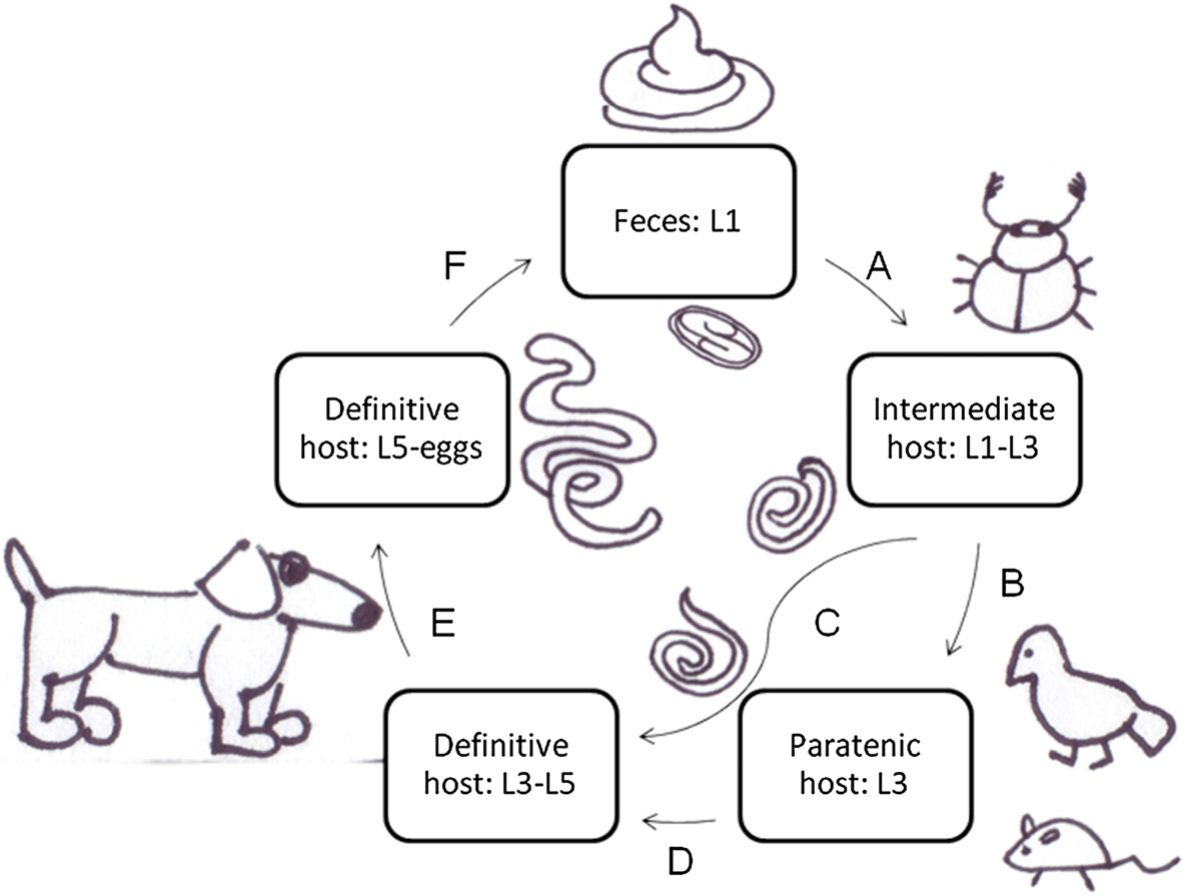
**Schematic life-cycle of *Spirocercal lupi*.** Eggs containing L1 larvae are found in the feces of the infected canid host (Feces: L1). The intermediate host, a dung beetle, consumes the feces and ingest the eggs (**A**). The eggs hatch and the larvae develop into L3 (Intermediate host: L1-L3). The intermediate host can either be consumed by paratenic hosts such as birds or small mammals (**B**), in which L3 arrest their development (paratenic host: L3), or by the definitive host (**C**) where the L3 larvae are released in the stomach, penetrate the gastric mucosa and migrate within blood vessel walls to the caudal thoracic aortic wall, where they develop to L4. From there, larvae migrate to the caudal esophagus, where they mature and sexually reproduce (**E**, Definitive host: L3-L5). Alternatively, the definitive host preys on L3 infected paratenic hosts (**D**). Adult worms are found in the esophageal wall, surrounded by a nodule. The female worms pass their eggs to the gastrointestinal tract, and into the feces (**F**, Definitive host: L5-eggs).

The definitive canid host of *S. lupi* is infected by ingesting an obligate intermediate coprophagous beetle vector, or a variety of paratenic hosts including birds, reptiles, amphibians and small mammals [10] that are infected by *S. lupi* (Figure 1). The main vector of *S. lupi* in Israel is the scarab beetle *Onthophagus sellatus* (Coleoptera: Scarabidae) [11]. The beetle ingests *S. lupi* eggs upon feeding on the definite host's feces, and within the beetle intermediate host, the infective larvae (L3) develop. Upon ingestion of the beetle, or the paratenic host, by the definitive host, L3 are released in the stomach, penetrate the gastric mucosa and migrate within blood vessel walls to the caudal thoracic aortic wall, where they develop to L4. From there, larvae migrate to the caudal esophagus, where they mature and sexually reproduce. In the esophageal wall the nematodes are surrounded by a nodule, comprised of fibroblasts. The female worms burrow a tunnel through the esophageal wall and pass their eggs, which contain larvae (L1) to the gastrointestinal tract, and into the feces. Dogs infected by *S. lupi* present variable clinical signs, depending on the stage of the disease. The esophageal nodule can undergo neoplastic transformation, resulting in development of sarcomas (Reviewed in 9). In Israel, spirocercosis is an emerging disease since the 1990's, with 50 dogs diagnosed with the disease annually at the Hebrew University Veterinary Teaching Hospital (HUVTH), most from the Greater Tel Aviv area [8]. Since then, the geographic distribution disease in Israel has widened, and during 2009, 91 dogs were diagnosed with spirocercosis at the HUVTH, of which 33 dogs had neoplastic esophageal disease, and died or were euthanized shortly post presentation. Additionally, the geographic distribution of the disease during this period had widened, and is no more restricted to the Greater Tel-Aviv area, but includes all the subtropical areas in the country (I. Aroch, unpublished data).

Diagnosis of spirocercosis is always challenging, because the clinical signs are variable and occur in advanced disease stages. Most animals are thus diagnosed only in the advanced stage of the disease, once nodules containing adult to egg shedding worms, are present in the esophagus [8]. The diagnosis of the disease in its early stages, prior to formation of esophageal nodules and egg shedding, is currently difficult and is almost impossible.

Recent studies have shown a relationship between bacterial symbionts of the genus *Wolbachia* and filarial pathogenic nematodes [12]. *Wolbachia* which is estimated to infect 66% of arthropods and nematodes [13] can manipulate various aspects of its arthropod hosts’ biology [14]. *Wolbachia* was found to be an obligatory symbiont of certain filarial nematodes, with a possible role in the pathogenesis and immune response to filarial infection in the mammalian host [4,5,15,16].

In the current study, we tested for the presence of *Wolbachia* species and other specific symbionts in the nematode *S. lupi*, and detected a novel and stable infection in the worm. Our findings are expected to promote further understanding of the interactions among various organisms in complex systems such as spirocercosis, and may have clinical implications, because this stable bacterial infection can potentially be used for novel simple diagnostic methods of this disease and aid in its prevention and treatment.

## Results and discussion

### Identification of novel bacterial symbiont in *S. lupi* from the Thelazioidea super family

**Figure 2:**
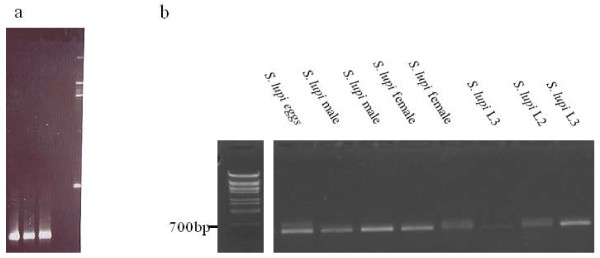
**Detection of a single bacterium, *Comamonas *sp., in *Spirocerca lupi *(a)** Separation of DNA samples from 3 adult *S. lupi* after PCR analysis with general eubacterial primers on denaturing gradient gel electrophoresis (40% to 60% urea/formamide gradient) showing a single band result. (**b**) Detection of *Comamonas* sp. in DNA samples of *S. lupi* eggs, larvae (L2, L3), and adults (males and females), using PCR with *Comamonas* sp. specific primers.

DNA of *S. lupi* adults and larvae was extracted as described below, and was used for the detection of possible bacterial symbiont species including *Wolbachia*, *Cardinium* and *Rickettsia*, by diagnostic PCR using specific primers. All *S. lupi* DNA samples were found to be negative for these bacteria, while all the control DNA samples were positive, as expected. This is in agreement with other studies, that have failed to detect *Wolbachia* in certain species of the super family Filarioidea [17], and in other previously tested non-filarial nematode groups ([18] and reference within). Thus, in order to detect other possible bacteria within the nematode, general 16S rDNA (*rrs* gene) primers able to detect most known Eubacteria were used in PCR. Adult nematode's DNA templates were positive for this bacterial gene, and the PCR products were cloned and sequenced. BLAST analysis (http://blast.ncbi.gov.il/) revealed initial similarity to sequences of the genus *Comamonas*, a beta-Proteobacterium of the Comamonadaceae family, as published in GenBank. Consensus sequence of the identified *Comamonas* sp. was determined, and deposited in GenBank under the accession number: JQ361660. In addition, for detection of other bacteria, *rrs* PCR-DGGE analysis was performed. DGGE separation resulted in a single product, suggesting that *S. lupi* probably carries only a single bacterial species (Figure 2a). Sequences of the excised DGGE band were also highly similar to the genus *Comamonas*. Based on the consensus sequence, *Comamonas* sp. specific primers were designed and used in nested PCR on DNA template extracted from *S. lupi* adults, larvae (L2 and L3) and eggs. All these stages were found to carry the same *Comamonas* bacterium (Figure 2b). These findings suggest that this novel *Comamonas* sp. is vertically transmitted, and suggests that a long-term association between *Comamonas* sp. and *S. lupi* exists.

### Phylogenetic analysis of the *S. lupi* symbiont

**Figure 3:**
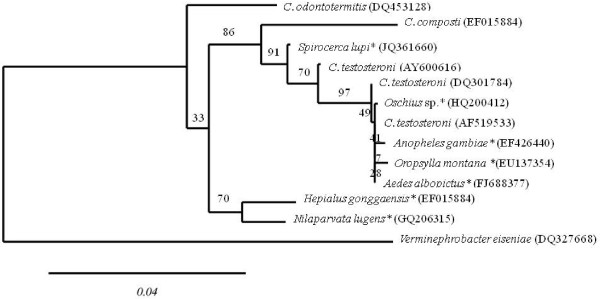
***Comamonas *sp. from *Spirocerca lupi *is closely related to soil derived *Comamonas *spp. and to *Comamonas *spp. from blood feeding arthropods.** Phylogenetic analysis based on maximum likelihood tree (1000 bootstraps) constructed with 16S rDNA sequences of various *Comamonas* species from different origin and host species. Host species are marked with asterisks. Published GenBank accession numbers are noted for each species. Bootstrap values are indicated on branches.

Based on a nearly full length *rrs* gene from the above identified *Comamonas* symbiont of *S. lupi*, and other selected *Comamonas* spp. sequences, a phylogenetic tree was built. The phylogeny analysis showed that the current *Comamonas* sp. sequence is clustered in a separate branch, together with *C. testosteroni*, known to participate in steroid degradation [19], and other soil-derived *Comamonas* species, represented herein by *C. composti *[20] (Figure 3). *Comamonas* spp., however, are not strict soil bacteria, and have recently been described in several insect species. Interestingly, the *S. lupi*-dervied *Comamonas* sp. is clustered in the same clade of *Comamonas* spp. identified in blood feeding insects, such as mosquitoes [21,22] and a flea [23]. This clade is separated from *Comamonas* spp. identified in non-blood feeders, namely the termite *Odontotermes formosanus *[24], a plant hopper, and a moth (Su and Li 2010: GenBank report GQ206315, Yin et al. 2008: GenBank report EU344924, respectively). The same clade also includes a *Comamonas* sp. identified in a soil nematode, *Oscheius* sp. (Deepa et al. 2010: GenBank report HQ200412). None of these studies, however, have suggested a role for these *Comamonas* spp. in their invertebrate hosts.

At present, the role that the identified *Comamonas* sp. plays in the biology of the nematode remains unknown, and so is its potential role in canine spirocercosis. A recent study, however, showed that benign infection with *S. lupi* induces an immune response that is atypical to chronic helminthic infection, but rather suggests a bacterial infection [25].

### Localization of *Comamonas* sp. within *S. lupi*

**Figure 4:**
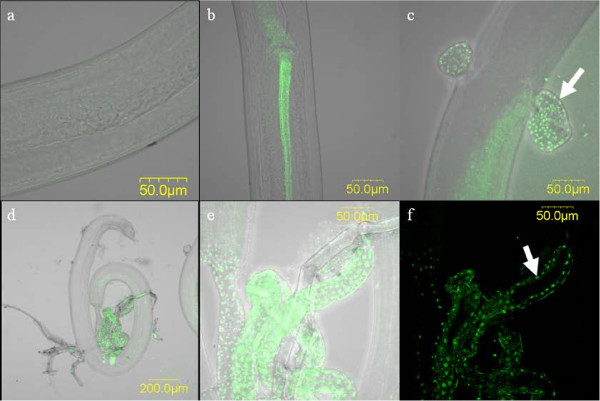
***Comamonas *sp. is restricted to the gut epithelium of *Spirocerca lupi *L3 larva.** Images of fluorescence *in-situ* hybridization analysis of *S. lupi* L3 larva stained with *Comamonas*-specific probe (green), detected using confocal microscopy. (**a**) No-probe control; (**b**) Intact L3; (**c,d**) Ruptured L3; (**e**) Enlargement of (**d**), showing specific signal in the larval gut; (**f**) One optical section showing a specific signal only in the gut epithelium region. The arrow points to a specific focal point. All images but (**f**) are combined optical Z sections, overlaid on a bright-field image.

Based on the *rrs* sequence of the novel *Comamonas* sp., a specific probe for fluorescent *in situ* hybridization (FISH) analysis was designed. Using the *Comamonas*-specific probe, we were able to demonstrate a specific signal in the gut epithelium of *S. lupi* larvae (Figure 4). The localization of the present *Comamonas* bacterium in the nematode's gut epithelium, and the phylogenetic proximity to other *Comamonas* spp. detected in blood-feeding insect hosts, may suggest that this novel *Comamonas* sp. plays a role in blood digestion or degradation within *S. lupi*, which feeds on its vertebrate hosts’ blood and tissues [9]. In addition, the FISH result, combined with the detection of *Comamonas* sp. in all the tested developmental stages of *S. lupi* using PCR, as described above, are in support of a stable, non- axenic infection of *S. lupi* by this bacterium.

### Detection of *S. lupi*-derived *Comamonas* sp. in blood samples of infected dogs

**Figure 5:**
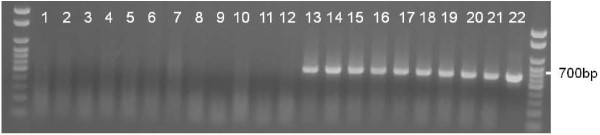
**DNA of Comamonas sp. can be detected in blood samples of dogs infected with *Spirocerca lupi*.** PCR detection of *Comamonas* sp. in samples of DNA extracted from blood of dogs infected with *S. lupi*. 1-no template control, 2-*Trichinella spiralis*, 3-healthy dog, 4-21-sick dogs, 22- *S. lupi* L3.

DNA detection from the *S. lupi*-derived *Comamonas* sp. in infected dogs may potentially be important in understanding the pathogenesis and promoting the diagnosis of spirocercosis. Recently, the symbiotic bacterium *Wolbachia* was detected in blood samples of dogs infected by the heartworm *Dirofilaria immitis *[26]. In the present study, we used a diagnostic semi-nested PCR with *Comamonas*-specific primers on DNA extracted from blood samples of dogs definitely diagnosed with spirocercosis and of negative control dogs. *Comamonas* sp. DNA was detected in 9/18 (50%) samples obtained from dogs with spirocercosis, but in none of 11 negative control samples (Figure 5). The rather low detection rate of *Comamonas* sp. in the dogs infected with the nematode may be due to several reasons; an unavailable bacterial template; improper storage of blood samples, resulting in insufficient DNA preparation, or an undetectable symbiont template in standard PCR due to unknown PCR inhibitors on a low concentration of *Comamonas* DNA in the blood. Alternatively, detection of the symbiont in blood samples may depend on the specific interactions between the bacterium and the nematode within the definitive canine host. It may be speculated that bacteria are only released from the nematode upon its death and disintegration, or within a limited specific time-point during infection within the definitive canine host. Further studies are warranted, to assess the optimal blood storage protocols and DNA extraction methods of canine samples, along with spiking experiments with *Comamonas* sp. and quantitative PCR in order to devise an efficient molecular diagnostic protocol.

## Conclusions

In the present study, we detected an additional organism, a bacterial symbiont of the genus *Comamonas*, within the causative agent of spirocercosis, the nematode *S. lupi*. Recently, microbial symbiosis has been repetitively shown to be a driving force in the biology and evolution of many organisms. The present study adds yet additional evidence of this trend, in a highly complex system. Resolution of the complex interactions among the different organisms involved in the spirocercosis system may lead to novel, applicable methods for the early diagnosis, prevention and treatment of canine spirocercosis, in a similar manner as has been applied when the interaction between *Wolbachia* spp. symbionts with their filarial nematode hosts has been elucidated [3, http://a-wol.com].

## Methods

### Sample origin

Adult *S. lupi* worms were obtained from esophageal nodules of dogs diagnosed with spirocercosis at the Hebrew University Veterinary Teaching Hospital, at necropsy, and stored in −20°C pending analysis. Larvae (L2 and L3) were dissected under a stereoscope from *O. sellatus* beetles, isolated in the laboratory from dog fecal dungs, collected in a public park located in a *S. lupi*-endemic area in Central Israel [11]. These were either stored in absolute ethanol at −20°C, or freshly used. *S. lupi* eggs were concentrated through floatation [27], and stored as described above.

Blood samples were obtained from dogs diagnosed with spirocercosis through esophageal endoscopy and presence of eggs in the feces, and from puppies aged 2 to 4 months, housed in a breeding farm. Puppies were chosen as negative control because they were housed in a restricted kennel, and were thus unexposed to feces of other dogs.

### DNA extraction, PCR, clone library and sequencing

DNA of adult *S. lupi* worms was extracted using hexadecyltrimethyl ammonium-bromide (CTAB) buffer [28], and were used in PCR with the 16S rDNA (*rrs*) gene primer set, targeting most known eubacteria (27F-1494R; [29]), under the following reaction conditions: 3 min at 95°C; 35 cycles of 1 min at 95°C, 1 min at 55°C, 1.5 min at 72°C; and 5 min at 72°C. The PCR products were run on 1% agarose gel, and were later extracted and cloned into pGEM-T easy vector (Promega, Madison, WI, USA), and transformed into competent *Escherichia coli*. Plasmids from 10 inserted clones were extracted from the gel and sequenced (HyLabs, Rehovot, Israel). As a control for DNA quality, PCR analysis was performed using primers for the *S. lupi*-specific cytochrome oxidase subunit 1 gene (*cox1*) as previously described [30].

### Direct probing of known invertebrate symbiont

DNA of *S. lupi* was used in PCR with specific primers and conditions to identify *Wolbachia*, *Cardinium* and *Rickettsia* spp., as previously described [31]. DNA extracted from *Bemisia tabaci*, harboring *Wolbachia* and *Rickettsia* spp., and from *Plagiumerus diaspidis* containing *Cardinium* sp. were used as positive controls.

### Denaturating gradient gel electrophoresis (DGGE)

PCR was performed on adult *S. lupi* DNA using primers GC-clamp 341 F-907R, targeting the bacterial *rrs* gene, with PCR conditions permitting its amplification from most known bacteria [32]. DGGE was performed using a 40% to 60% urea/formamide gradient for standard reactions. After the electrophoresis, gel was incubated in ethidium-bromide solution (250 ng/ml) for 10 min, rinsed in distilled water, and photographed under UV illumination. Bands were extracted, and sent for direct sequencing (HyLab, Rehovot, Israel).

### Phylogenetic analysis

Nine nearly-full length sequences of the *rrs* gene of *Comamonas* sp. were obtained from five adult *S. lupi* worms. All clones were sequenced from both directions. Sequences were edited using DNAMAN software (Lynnon Corporation, Canada) and a consensus sequence was determined. The *Comamonas* sp. *rrs* sequence was aligned, using MUSCLE 3.7, with other published *Comamonas* spp. sequences, selected based on BLAST results, and based on their invertebrate host origin. The *rrs* gene sequence of *Verminephrobacter eiseniae* was used as an out group. A maximum-likelihood tree was constructed using PhyML 3.0 software. Bootstrap analyses with 1000 re-samplings were performed to test branching robustness. The tree was illustrated using TreeDyn 198.3. All software packages are available at http://www.phylogeny.fr/.

### Direct probing of *Comamonas* sp.

To confirm the presence of *Comamonas* sp. in the various *S. lupi* developmental stages (eggs, larvae and adults), and in blood samples obtained from *S. lupi-*infected dogs, a diagnostic PCR was planned. Based on the *rrs* sequence established, specific primers were designed; /ComF323/ 5’-CCTCGGGTTGTAAACTGCTT-3’ and /ComR1393/ 5’-TCTCTTTCGAGCACGAATCC-3’. The primers were used in a standard PCR, under the following conditions: 3 min at 95°C; 35 cycles of 1 min at 95°C, 1 min at 58°C, 1 min at 72°; and a final 5 min at 72°C. The PCR product size was expected to be ca. 1000 bp. Positive and negative PCR products were retested using semi-nested PCR, with the forward primer /ComNest F/ 5’- ACTGCCATTGTGACTGCAAG-3’ and the ComR1393 reverse primer, with PCR conditions as described above, resulting in ca. 600 bp product. Three PCR products from each sample category were directly sequenced in order to confirm the *Comamonas* specific sequence.

### Fluorescent *in-situ* hybridization (FISH)

FISH was performed as previously described [33]. Briefly, larvae were fixed in Carnoy’s fixative (6:3:1 parts of chloroform: ethanol: acetic acid), and later hybridized with the *rrs*-based designed probe: Com-probe /Cy3/ 5’- TGTGCTACTAGAGCGGCTGA-3’, in hybridization buffer. Since intact larvae could not uptake the probe, larvae were first ruptured using sterile insect pins, and their content was removed from the cuticle. Specimens were viewed under an IX81Olympus FluoView500 confocal microscope. Signal specificity was confirmed based on sequence comparison in the ‘Probe Match’ function in the Ribosomal Database Project website (http://rdp.cme.msu.edu/), and using a no-probe control, and hybridization to a non-target nematode, *Trichinella spiralis*.

### Ethics statement

Samples (nematodes and blood) were obtained from *S. lupi*-infected dogs presented to the Hebrew University Veterinary Teaching Hospital, Koret School of Veterinary Medicine, Hebrew University of Jerusalem with their owners' consent, during diagnosis, treatment and necropsy. Samples obtained from control dogs were obtained with their owner's consent. This study was approved by the Institutional Committee of Animal Handling and Experimentation.

## Competing interests

The authors declare that they have no competing interests.

## Authors’ contributions

YG conceived the study, participated in its design, performed the molecular identification as well as phylogenetic and FISH analyses, and wrote the paper. MK performed the specific molecular detection, and analyzed the data. AM participated in the study design and perform the eggs and larvae collections. MG participated in the study design, provided controls and drafted the paper. EL and IA conceived the study, participated in its design, provided adult worms, and drafted the paper. All authors read and approved the final manuscript.
